# 

**Published:** 1892-02

**Authors:** 


### TABLE OF CONTENTS.

ORIGINAL COMMUNICATIONS. PAGE

The Management of Dental Practice. By LOUIS JACK, D.D.S., Philadelphia . . . 81

Crown- and Bridge-Work. By FREDERIC A. PEESO, D.D.S., Philadelphia 90

On the Value of Crystal Gold in Dentistry. By N. W. WILLIAMS, D.D.S., Nice, France . . . 100

Saw-Frame and Gum-Guard. By DR. W. H. ROLLINS . . . 102

REPORTS OF SOCIETY MEETINGS.

New York Odontological Society . . . 103

American Dental Association . . . 115

American Academy of Dental Science . . . 126

Odontological Society of Pennsylvania . . . 136

Central Dental Association of Northern New Jersey . . . 142

EDITORIAL.

To Our Subscribers . . . 151

In Response . . . 152

BIBLIOGRAPHY . . . 154

CURRENT NEWS.

Odontographic Society of Chicago . . . 155

Annual Meeting of the Louisiana State Dental Society . . . 156

SELECTIONS.

Carbolate of Camphor . . . 156

Physiological Action of Peroxide of Hydrogen . . . 157

Carbolic Acid, Creolin, and Lysol . . . 158

Mercurial Stomatitis . . . 159

The Struggle against Disease . . . 160

